# Microalbuminuria and sRAGE in High-Risk Hypertensive Patients Treated with Nifedipine/Telmisartan Combination Treatment: A Substudy of TALENT

**DOI:** 10.1155/2012/874149

**Published:** 2012-02-13

**Authors:** Colomba Falcone, Maria Paola Buzzi, Sara Bozzini, Chiara Boiocchi, Angela D'Angelo, Sandra Schirinzi, Ciro Esposito, Massimo Torreggiani, Jasmine Choi, Michael Ochan Kilama, Giuseppe Mancia

**Affiliations:** ^1^Interdepartmental Center of Research in Molecular Medicine (CIRMC), University of Pavia, 27100 Pavia, Italy; ^2^Department of Cardiology, Istituto di Cura Città di Pavia, University Hospital, 27100 Pavia, Italy; ^3^Department of Nephrology, IRCCS Policlinico San Matteo, University of Pavia, 27100 Pavia, Italy; ^4^Scientific Relations, Medical Department, Bayer S.p.A-Pharmaceutical, 20156 Milan, Italy; ^5^Department of Clinical Medicine and Prevention, University of Milano-Bicocca, 20126 Milan, Italy

## Abstract

Some antihypertensive drugs have also renoprotective and anti-inflammatory properties that go beyond their effect on blood pressure. It has been suggested that microalbuminuria and glomerular filtration rate (GFR) are associated with circulating levels of the soluble form of the receptor, sRAGE (soluble receptor for advanced glycation ends-products). In the present analysis, we used data from the TALENT study to evaluate soluble receptor for advanced glycation end-products (sRAGE) plasma levels in patients with hypertension and high-cardiovascular risk-treated nifedipine and telmisartan in combination. Treatment with nifedipine-telmisartan significantly decreased mean systolic and diastolic ambulatory blood pressure and resulted in a significant increase in sRAGE plasma concentrations after 24 weeks of therapy. We concluded that in hypertensive patients with early-stage renal disease, sRAGE concentrations are not influenced by either microalbuminuria or GFR. Long-term treatment with a combination of nifedipine-telmisartan may have a beneficial effect increasing sRAGE plasma levels, thus exerting an atheroprotective and anti-inflammatory activity.

## 1. Introduction

Atherosclerosis has been regarded recently as an inflammatory disease. Most cardiovascular risk factors are associated with enhanced vascular inflammatory state [1]. Among these, hypertension has been suggested to have a proinflammatory action by inducing endothelial expression of cytokines [2]. C-reactive protein (CRP), leukocyte adhesion molecules, chemotactic and pro-inflammatory cytokines and heat shock proteins were found to be increased in patients with essential hypertension [3, 4]. Hypertension and cardiovascular disease (CVD) are intimately connected to chronic kidney disease (CKD) [5]. High blood pressure has also been associated with the development of end-stage renal disease [6]. On the other hand, atherosclerosis, considered one of the traditional risk factors for CVD, is more prevalent in CKD patients, at any stage, than in the general population [7]. Several observational studies have suggested that some antihypertensive drugs such as the inhibitors of the renin-angiotensin system (RAS) or dihydropyridine-based calcium antagonists promote normal endothelial function, thus showing anti-inflammatory activity [8, 9]. Due to this specific anti-inflammatory property, the RAS blockade can exert another effect, independently of its hemodynamic action, namely, the reduction of the development and progression of kidney disease [10]. In the setting of nephroprotection, the beneficial effect of counteracting the RAS system has been suggested to be due, at least in part, to the modulation of the interaction between the receptor for advanced glycation end products (RAGE) and its ligands [11]. RAGE is a multiligand member of the immunoglobulin superfamily of cell surface molecules, which was first described as a receptor for advanced glycation end products (AGEs) [12, 13]. AGEs have a proinflammatory action, and have been related to endothelial dysfunction, arterial stiffening and hypertension [14]. Furthermore, the interaction between RAGE and its ligands is intimately involved in the pathobiology of a wide range of diseases that share common features, such as enhanced oxidative stress, immune/inflammatory responses, and impaired cell functions [15]. RAGE has a secreted isoform, termed soluble RAGE (sRAGE) that lacks the transmembrane anchoring domain and therefore circulates in plasma. Soluble RAGE has AGE-binding properties and acts as a scavenger receptor, preventing the generation of the signalling cascade usually associated with RAGE, thus acting as an inhibitor of AGE-mediated effects [16]. High plasma levels of sRAGE have been reported to be associated with a lower incidence of coronary artery disease (CAD) [17], and low plasma levels of sRAGE have been reported in patients with carotid and femoral atherosclerosis as well as those with metabolic syndrome [18]. Recently, it has been found that sRAGE correlates with the extent of 24-hour albumin excretion, confirming the involvement of the RAGE-ligand axis in the development of diabetic nephropathy [19]. Nevertheless this correlation has not been confirmed by subsequent studies on patients with normal values of glomerular filtration rate (GFR) and therefore the role of sRAGE in the setting of early-stage renal disease has not been fully elucidated [20]. Moreover, a number of drugs recently proved to be able to modulate sRAGE levels [21].

The aims of the present study were (1) to study the association between sRAGE and microalbuminuria in hypertensive patients at high cardiovascular risk and (2) to evaluate the effect of treatment with telmisartan and nifedipine, which are antihypertensive drugs with specific anti-inflammatory effect, on sRAGE levels according to the presence or absence of microalbuminuria.

## 2. Methods

### 2.1. Study Population

Participants were derived from the TALENT study (multicenter study evaluating the Efficacy of nifedipine GITS-telmisartan combination in blood pressure control and beyond). TALENT was a multicenter randomized double-blind prospective parallel group (3-arms) trial. It was approved by Ethics Committees/Institutional Review Boards of the centers involved and was conducted according to Good Clinical Practice and under the guiding principles of the Declaration of Helsinki. All individuals provided their informed consent to participate. Included were men and women aged 18–75 years whose office systolic blood pressure was higher than 135 mmHg and with one or more of the following cardiovascular risk factors: type 2 diabetes mellitus (fasting blood glucose ≥126 mg/dL or history of antidiabetic drug use), microalbuminuria, electrocardiographic or echocardiographic evidence of left ventricular hypertrophy and metabolic syndrome. The protocol details and main study results are published elsewhere [22]. Patients with a history of stroke, a transient ischemic attack or myocardial infarction within the previous 12 months, those on angiotensin receptor antagonist or calcium antagonist and those with renal failure (serum creatinine >2 mg/dL) or macroalbuminuria were excluded from the study. Patients were randomized to one of the following treatment groups in a 2 : 1 : 1 ratio: group A received the combination of nifedipine GITS 20 mg/telmisartan 80 mg per day; group B received monotherapy with nifedipine GITS 20 mg/day; group C received monotherapy with telmisartan 80 mg/day. After 8 weeks of treatment, all the 2 monotherapy groups were switched to the combination (telmisartan 80 mg-nifedipine GITS 20 mg per day). From week 16 to week 24, patients were allowed to continue the combination treatment for an additional 8 weeks with the option to uptitrate nifedipine GITS to 30 mg.

To limit any potentially confounding effects due to outlying values, we chose to exclude from our analysis subjects with any CRP values >10 mg/L. We also excluded those with missing serum biomarkers or microalbuminuria analyses at any of the study visit.

### 2.2. Methods

Frozen blood sample from baseline, 16 and 24 weeks, were simultaneously analyzed at a core facility. As in previous clinical studies involving total sRAGE (esRAGE plus cRAGE), we determined plasma sRAGE levels using a commercially available ELISA kit (Quantikine; R&D systems) according to the manufacturer's instructions. Briefly, a monoclonal anti-sRAGE antibody was used to bind plasma sRAGE, and bound sRAGE detected with a horseradish-peroxidase-linked polyclonal antibody specific for the extracellular domain of RAGE. After washing, a hydrogen peroxide solution was added to each well, and optical density at 450 nm was determined with a microplate reader (Multiscan Ex, Thermolabsystems). The intraassay and inter-assay coefficients of variation values were 6% and 8%, respectively and the minimum detectable dose of RAGE ranged from 1.23–16.14 pg/mL. The presence/absence of microalbuminuria was detected by local laboratories by the CLINITEK Status Analyzer, Siemens. GFR was estimated using the Modification of Diet in Renal Disease Study equation, equation 7, derived by Levey et al. [23].

All measurements were performed without knowledge of the treatment assignment.

### 2.3. Statistic

Continuous data are reported as mean ± SD along the paper, even in included tables, if the data were skewed. Categorical data are presented as per group percentages. Differences between subgroups were evaluated using Student's *t*-test for the normally distributed ones. If data were skewed, the logarithmic transformation was done to make the data valid for Student's *t*-test. Differences in categorical data were compared using the chi-square test. Simple (univariate) linear regression model was adopted to analyze the association of sRAGE with GFR. A two-tailed *P* value less than 0.05 was considered to be statistically significant. The relationship between two variables was tested using the Pearson's correlation coefficient.

## 3. Results

Among the 405 subjects included in the TALENT study, inflammatory markers and microalbuminuria data were available for 262 patients (173 males and 89 females, mean age  59 ± 9  years). Among those patients, 99 (38%) had a positive result at the microalbuminuria test (30–300 mg/24 h), while the remaining 163 (62%) had a negative result (<30 mg/24 h). The mean GFR value of our cohort at the recruitment was 93 ± 23 mL/min.

No significant differences were found in plasma sRAGE levels concerning sex of the participants (141 pg/mL versus 138 pg/mL, *P* = ns), according to the diabetic (134 pg/mL versus 152 pg/m) and smoking status (139 pg/mL versus 142 pg/mL, *P* = ns) or statin use (145 pg/mL versus 140 pg/mL, *P* = ns). The log-transformed sRAGE levels showed a positive correlation with serum creatinine (*r* = 0.14, *P* = 0.0059) and uric acid (*r* = 0.122, *P* = 0.016), but were not significantly correlated with systolic or diastolic blood pressure (*r* = 0.09 and *r* = −0.05, *P* = ns), GFR (*r* = −0.07, *P* = ns), and hsCRP (*r* = 0.05, *P* = ns) in the entire study cohort.

Baseline characteristics of the patients, according to the presence/absence of microalbuminuria are listed in Table 1. As shown in the Table 1, patients with and without microalbuminuria were similar with respect to age, sex, blood pressure, lipid profile, and GFR values. Plasma concentrations of sRAGE were similar between the two groups. Of interest, baseline plasma concentration of sRAGE was not associated with GFR (*r* = −0.08, *P* = ns), both in patients with (*r* = 0.11, *P* = ns) and without microalbuminuria (*r* = −0.06, *P* = ns).

After 16 weeks of telmisartan-nifedipine treatment according to the study protocol, a significant reduction in ambulatory blood pressure was observed: 24-h mean systolic blood pressure decreased from 137 ± 5 mmHg to 126 ± 5 mmHg (*P* < 0.001), and diastolic blood pressure decreased from 82 ± 5 mmHg to 75 ± 5 mmHg (*P* < 0.001). At that time point, the percentage of patients with microalbuminuria decreased from 38% to 29% (*P* < 0.001), and a trend towards an increase in sRAGE plasma concentration was observed (*P* = ns).

After other 8 weeks of treatment with the combination telmisartan-nifedipine, the percentage of patients with microalbuminuria decreased to 28% (*P* < 0.01 from baseline). Treatment with telmisartan-nifedipine resulted in a significant increase in sRAGE values as compared with baseline (log-transformed sRAGE 5.16 ± 0.6 versus 5.13 ± 0.6, *P* < 0.05). A similar increase in sRAGE plasma concentrations was found in patients with and without microalbuminuria (0.03 ± 0.2 versus 0.05 ± 0.2  *P* = ns).

**Figure 1:**
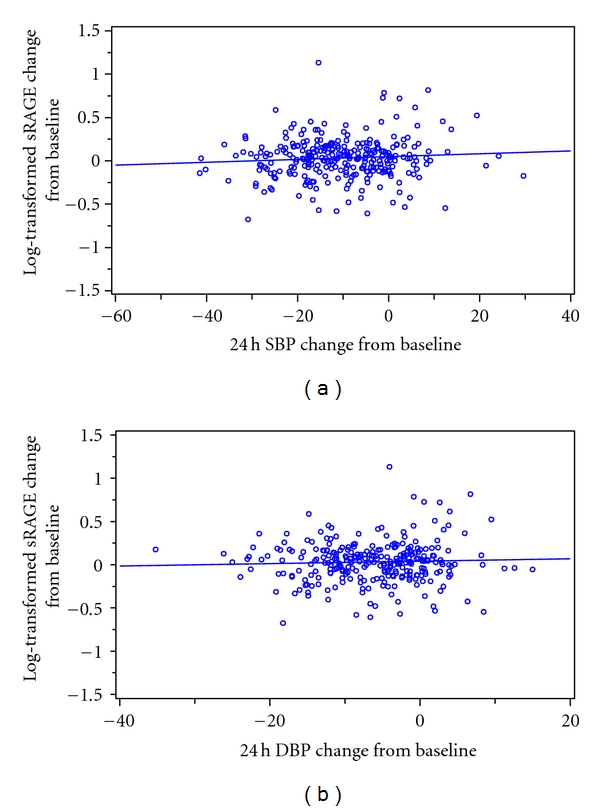
Log-transformed sRAGE by 24 hour systolic (a) and diastolic (b) blood pressure change from baselin at week 24.

Furthermore, we studied the relationship between the change in sRAGE values and ambulatory blood pressure from baseline to week 24. We observed that the increase in log transformed sRAGE concentrations was not proportional to the decrease in 24 h mean systolic nor diastolic blood pressure, as shown in Figure 1.

## 4. Discussion

According to the 2010 United States Renal Data System report, CKD patients, with any stage of CKD, have higher rates of hypertension, and the prevalence of hypertension increases with the stage of kidney disease [24]. Cardiovascular disease, as well, is tightly bound to impairment of kidney function, as demonstrated by Anavekar and colleagues in the VALIANT study, being the risk of death from any cardiac event inversely associated with the glomerular filtration rate, making even mild kidney dysfunction a major unconventional risk factor for cardiovascular complications [25]. RAGE signalling has been suggested to play a pivotal role in inducing inflammatory process and endothelial dysfunction, that characterize both diabetic and nondiabetic atherosclerosis and its clinical manifestations [15]. Microalbuminuria, which is an independent risk factor for renal and cardiovascular disease, is a feature of hypertension, metabolic syndrome, and type I diabetes mellitus and is supposed to be associated with higher values of sRAGE [19, 26]. In a subanalysis of the TALENT study, we found that the presence of microalbuminuria does not affect baseline sRAGE plasma concentrations. Furthermore, antihypertensive treatment with the combination nifedipine-telmisartan increased sRAGE values regardless of the presence of microalbuminuria. Even if the TALENT study was not designed to evaluate effect of the intervention on sRAGE levels, we can hypothesize that this is related to a specific effect of nifedipine-telmisartan on endothelial function.

Angiotensin II activates NAD(P)H oxidase which stimulates the production of reactive oxygen species; this increases the function of the proinflammatory transcription factor nuclear factor-*κ*B which controls the expression of proinflammatory cytokines. Angiotensin II also stimulates growth factors, extracellular matrix protein synthesis, and matrix metalloproteinases, thereby promoting proliferative and fibrotic mechanisms involved in vascular remodelling. Telmisartan has an additional specificity of action that relates to a partial peroxisome proliferator-activated receptor-*γ* (PPAR-*γ*) agonism. In vitro studies have recently shown that through this peculiar function of PPAR-*γ*, telmisartan inhibits superoxide generation and monocyte chemoattractant protein-1 (MCP-1) gene expression in mesangial cells, thus exerting an anti-inflammatory activity against AGE-mediated kidney damage [27].

Long-acting CCBs exert antioxidant effects that protect LDL and membrane lipids from oxidation, this effect being independent of calcium channel modulation [9]. It has been found that nifedipine inhibits the TNF-*α*-induced generation of reactive oxygen species and the subsequent MCP-1 and VCAM-1 expression in human cultured endothelial cells by suppressing NADPH oxidase activity. Furthermore, nifedipine is known not only to upregulate endothelial nitric oxide synthase expression, but also to increase NO bioactivity via superoxide dismutase induction in endothelial cells [28].

As a consequence, both telmisartan and nifedipine share the potential to exert specific anti-inflammatory actions that go beyond their class effects. Evidences from preclinical and clinical studies suggest that the complementary mechanisms of action of these drugs provide greater efficacy when used in combination. TALENT confirms that the duration of treatment is a key factor: as a matter of fact, the increase in sRAGE was significant only after 24 weeks of treatment whereas blood pressure reduction was already evident after two weeks [22].

Our observation of a significant increase in sRAGE values during nifedipine-telmisartan treatment corroborates the possible role of these drugs in influencing RAGE-ligands signaling as already shown also in other studies [27, 29]. The biological consequence of an increased amount of endogenous sRAGE is an anti-inflammatory and antithrombotic effect. Interactions between RAGE and its ligands result in enhanced transcription and production of various pro-inflammatory mediators via activation of nuclear factor-*κ*B. These RAGE-dependent pro-inflammatory genes include intracellular adhesion molecule-1 (ICAM-1), VCAM-1, E-selectin, TNF-*α*, interleukin-1, interleukin-6, and cyclo-oxygenase-2. Therefore, ligand-RAGE binding is thought to be involved in the formation, progression, and instability of atherosclerotic plaques with subsequent macrovascular complications. Since sRAGE is the circulating isoform of RAGE which has been identified and theorized to competitively prevent the binding of AGEs to the transmembrane form of RAGE, drugs that increase sRAGE plasma concentrations may have the effect of attenuating atherosclerosis. This hypothesis has been supported by animal models in which administration of sRAGE to mice retarded the progression of atherosclerosis [30].

Microalbuminuria is a marker of microvascular endothelial dysfunction, and reduction of microalbuminuria could reflect a better haemodynamic situation, namely, a decreased shear stress, that ameliorates the endothelial performance and modifies sRAGE levels, thus reducing the cardiovascular risk [31]. In the TALENT study, we found that sRAGE plasma levels were not affected by the presence or absence of microalbuminuria. Our data are consistent with previous observations on diabetic Chinese patients with early stage-renal disease [32]. On the contrary, in patients with advanced degree of kidney dysfunction, documented by a reduced GFR and sRAGE concentrations were found to be positively associated with increased 24-hour albumin excretion [33]. The mean value of GFR in our study population was 93 mL/min, confirming that our patients can be considered at an early stage of renal disease, since patients with elevated creatinine plasma levels and those with macroalbuminuria (spot urine albumin/creatinine ratio >300 mg/g) were excluded from the study. Therefore, the apparent discrepancies between the studies could be explained by the different severity of renal impairment.

Our study did not find any associations between sRAGE and GFR values. A recent study by Basta and colleagues identified a GFR of approximately 60 mL/min as a threshold below which sRAGE accumulates [34]. Thus, the lack of association between GFR and sRAGE plasma levels in our study could be explained by the GFR higher than 90 mL/min.

The role of calcium antagonists on the kidney with previous formulations of the drugs [35, 36] has been quite controversial; however, more recent controlled trials, including those with nifedipine GITS, demonstrated an effective renal protection as long as blood pressure is also controlled [37]. In addition, a recently published paper by Nakamura and colleagues has demonstrated the beneficial effect of a calcium channel blocker on sRAGE levels in a small cohort of nondiabetic patients with early-stage kidney disease [38]. Our data are in line with the forementioned study and have the advantage of having been achieved on a larger population. The combination of a calcium antagonist and an angiotensin receptor blocker has a strong rationale and should be more effective than the monotherapies as demonstrated in the TALENT study [22].

A limitation of our study is that the assay used to assess sRAGE levels is unable to distinguish among different circulating isoforms of sRAGE. Therefore, since the proportion of different isoforms of sRAGE could differ among individuals, our study cannot clarify the pathophysiology of the regulation of sRAGE production in patients with and without microalbuminuria, which would be the subject of a different study.

In conclusion, our study suggests that in high-risk hypertensive patients plasma sRAGE levels do not correlate with microalbuminuria as long as the GFR is preserved. In this specific population, the increase of sRAGE plasma concentration and the reduction of microalbuminuria may indicate benefits that go beyond blood pressure reduction obtained by a nifedipine-telmisartan combined treatment.

## Figures and Tables

**Table 1:** Baseline characteristics of patients with and without microalbuminuria. Continuous data are reported as mean ± SD or median (interquartile range), categorical data are presented as per group percentages.

Variable	With microalbuminuria *N* = 99	Without microalbuminuria *N* = 163	*P* value
Age, years	60 ± 10	58 ± 10	ns
Males	62%	68%	ns
Diabetes mellitus	59%	61%	ns
Mean 24-h SBP, mmHg	137 ± 5	136 ± 5	ns
Mean 24-h DBP, mmHG	84 ± 5	85 ± 5	ns
Total cholesterol, mmol/L	5.06 ± 0.96	5.14 ± 1.14	ns
HDL cholesterol, mmol/L	1.24 ± 0.31	1.21 ± 031	ns
LDL cholesterol, mmol/L	3.1 ± 0.85	3.15 ± 1.01	ns
Triglycerides, mmol/L	1.74 ± 1.1	1.65 ± 0.95	ns
Body mass index, Kg/m^2^	29 ± 3	29 ± 3	ns
Smokers, %	56%	48%	ns
Creatinine, *μ*mol/L	82.2 ± 19.4	82.2 ± 19.4	ns
GFR mL/s/1.73 m^2^	1.55 ± 0.38	1.57 ± 0.45	ns
Log-transformed sRAGE	5.12 ± 0.6	5.14 ± 0.6	ns

SBP: systolic blood pressure; DBP: diastolic blood pressure; GFR: glomerular filtration rate.
